# P-2170. Detection of Human Adenovirus with and without Co-detection of Other Respiratory Viruses Among Children with Acute Respiratory Illnesses

**DOI:** 10.1093/ofid/ofaf695.2333

**Published:** 2026-01-11

**Authors:** Adam E Gailani, Tess Stopczynski, Herdi Kurnia., Olla R Hamdan, Haya Hayek, Varvara Probst, Laura S Stewart, Rangaraj Selvarangan, Jennifer E Schuster, Mary E Moffatt, Marian G Michaels, John Williams, Leila C Sahni, Vasanthi Avadhanula, Julie A Boom, Mary A Staat, Elizabeth P Schlaudecker, Christina M Quigley, Geoffrey A Weinberg, Peter G Szilagyi, Janet A Englund, Eileen J Klein, Aaron T Curns, Heidi L Moline, Ariana Toepfer, Andrew J Spieker, James Chappell, Natasha B Halasa

**Affiliations:** Vanderbilt University Medical Center, Nashville, TN; Vanderbilt University Medical Center, Nashville, TN; Vanderbilt University Medical Center, Nashville, TN; Vanderbilt University, Nashville, TN; Vanderbilt University Medical Center, Nashville, TN; Vanderbilt Univerisity Medical Center, Nashville, Tennessee; Vanderbilt University School of Medicine, Nashville, Tennessee; Children’s Mercy Hospital, Kansas City, Missouri; Children's Mercy Kansas City, Kansas City, MO; Children's Mercy Kansas City, University of Missouri Kansas City School of Medicine, Kansas City, Missouri; University of Pittsburgh/ CHP, Pittsburgh, Pennsylvania; University of Wisconsin, Madison, Wisconsin; Baylor College of Medicine and Texas Children's Hospital, Houston, Texas; Baylor College of Medicine, Houston, TX; Baylor College of Medicine, Houston, TX; Cincinnati Children's Hospital Medical Center, Park Hills, Kentucky; Cincinnati Children's Hospital Medical Center, Park Hills, Kentucky; Cincinnati Children's Hospital Medical Center, Park Hills, Kentucky; University of Rochester Sch Med & Dent, Rochester, New York; UCLA, Los Angeles, California; Seattle Children’s Hospital/Univ. Washington, Seattle, Washington; Seattle Children's Hospital and University of Washington School of Medicine, Seatte, Washington; Centers for Disease Control and Prevention, Atlanta, Georgia; US-CDC, Atlanta, Georgia; Centers for Disease Control and Prevention, Atlanta, Georgia; Vanderbilt University Medical Center, Nashville, TN; Vanderbilt University Medical Center, Nashville, TN; Vanderbilt University Medical Center, Nashville, TN

## Abstract

**Background:**

Human adenovirus (HAdV) is a common cause of acute respiratory illness (ARI) in children and is frequently detected with other viruses (co-detection). The effect of co-detection on HAdV ARIs is incompletely understood, and recently the COVID-19 pandemic has disrupted the seasonality of many respiratory viruses. We aimed to characterize the demographics of children with HAdV ARI and co-detection patterns from 2016 through 2023.

**Table 1.**
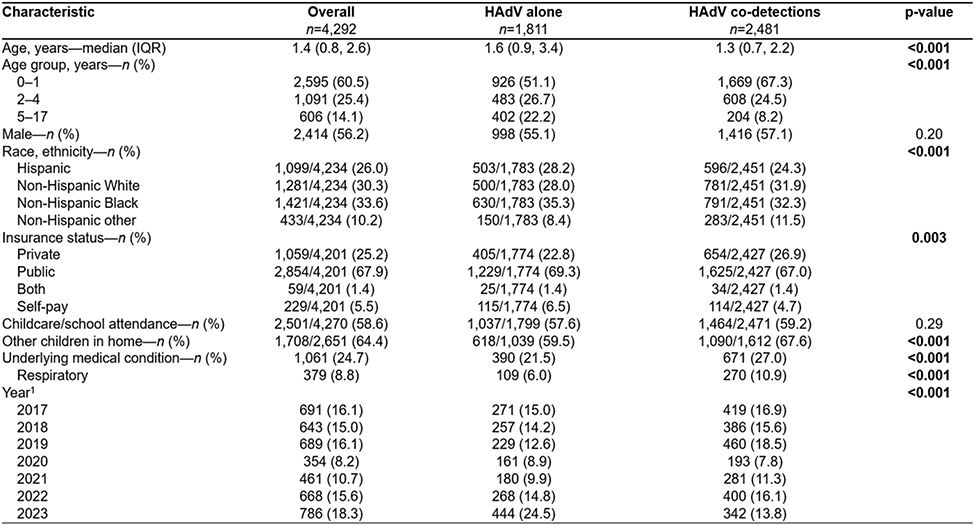
Demographic characteristics of children with HAdV, stratified by detection status: single HAdV detection vs. HAdV co-detected with one or more respiratory virus(es), December 2016 to August 2023, New Vaccine Surveillance Network. 1: The 2017 year includes cases from 12/01/2016 to 12/31/2017. The 2023 year includes cases from 01/01/2023 to 08/31/2023. We performed comparisons using Pearson’s χ2 test for categorical variables and the independent-samples t-test with unequal variances for continuous variables.

**Table 2.**
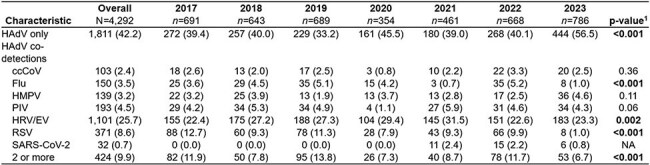
Single HAdV detection and HAdV co-detected with one or more respiratory virus(es), stratified by year, December 2016 to August 2023, New Vaccine Surveillance Network. Abbreviations: HAdV, human adenovirus; ccCoV, common cold coronaviruses; Flu, influenza; HMPV, human metapneumovirus; PIV, parainfluenza virus; HRV/RV, human rhinovirus/enterovirus; RSV, respiratory syncytial virus; SARS-CoV-2, severe acute respiratory syndrome coronavirus 2. 1Cells labeled NA indicate p-value could not be calculated due to zero cell counts. We performed comparisons using Pearson’s χ2 test for categorical variables and the independent-samples t-test with unequal variances for continuous variables.

**Methods:**

The New Vaccine Surveillance Network is an active, prospective, population-based ARI surveillance system at 7 US sites. Children < 18 years old with fever and/or ARI symptoms for < 14 days were enrolled if they were seen in the emergency department or admitted to the hospital. Nasal and/or oropharyngeal swabs were tested for respiratory viruses, and demographic information was collected. Demographics were compared between HAdV single detection and co-detections with other respiratory viruses, and the proportions of HAdV co-detections were compared between years.

**Figure 1.**
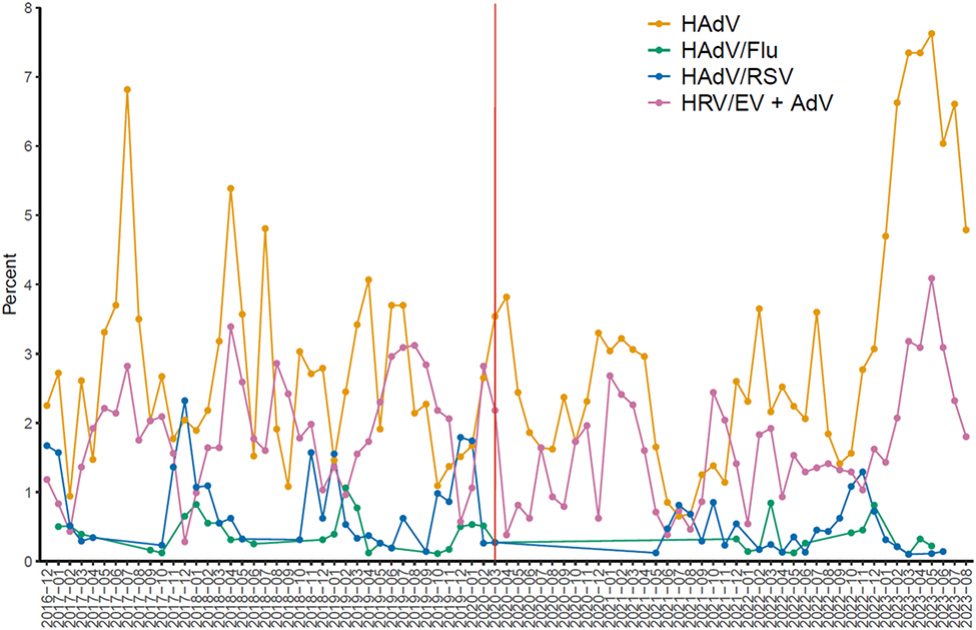
Seasonality of HAdV single detections and HAdV with other frequently co-detected viruses by percent of tested specimens, December 2016 to August 2023, New Vaccine Surveillance Network. Red line indicates when non pharmacologic interventions against SARS-CoV2 started in the US, March 2020

**Results:**

From Dec 2016 through Aug 2023, 4,292 of 66,662 (6.4%) children with ARI were positive for HAdV. 1,811 (42.2%) were single detections and 2,481 (57.8%) co-detections. Children with HAdV alone were older, had different racial/ethnic demographics, and were less likely to be privately insured, live with other children, or have an underlying respiratory condition (Table 1). The annual proportion of HAdV co-detections ranged from 43.5% to 66.8% (Table 2). The proportion of co-detections with specific viruses differed year to year for influenza, rhino/enterovirus, and respiratory syncytial virus, but not for common cold coronaviruses, human metapneumovirus, or parainfluenza virus. The variation and seasonality of HAdV-positivity and select co-detected virus fraction is shown in Figure 1.

**Conclusion:**

Most children with HAdV ARI had co-detection with another respiratory virus, with yearly variation in the proportion of co-detections and specific co-detected viruses. The demographics of children with HAdV single infection differed compared to those with co-detections, potentially representing varied exposure histories among different populations of children. These patterns could be relevant to HAdV ARI severity and inform management of HAdV infections.

**Disclosures:**

Rangaraj Selvarangan, PhD, Altona: Grant/Research Support|Biomerieux: Advisor/Consultant|Biomerieux: Grant/Research Support|Biomerieux: Honoraria|Cepheid: Grant/Research Support|Hologic: Grant/Research Support|Hologic: Honoraria|Meridian: Grant/Research Support|Qiagen: Grant/Research Support Marian G. Michaels, MD, MPH, Merck: Grant/Research Support Mary A. Staat, MD, MPH, Centers for Disease Control and Prevention: Grant/Research Support|Cepheid: Grant/Research Support|Merck: Advisor/Consultant|Merck: Grant/Research Support|National Institutes of Health: Grant/Research Support|Up-To-Date: Royalties Elizabeth P. Schlaudecker, MD, MPH, Gilead: Grant/Research Support|Pfizer: Grant/Research Support|Sanofi Pasteur: Advisor/Consultant Geoffrey A. Weinberg, MD, Inhalon Biopharma: Advisor/Consultant|Merck & Co: Honoraria Janet A. Englund, MD, AstraZeneca: Board Member|AstraZeneca: Grant/Research Support|Cidarra: Member Data Safety Monitoring Board|GlaxoSmithKline: Advisor/Consultant|GlaxoSmithKline: Grant/Research Support|Meissa Vaccines: Advisor/Consultant|Merck: Advisor/Consultant|Merck: Grant/Research Support|Moderna: Advisor/Consultant|Moderna: Grant/Research Support|Pfizer: Advisor/Consultant|Pfizer: Grant/Research Support|Shionogi: Grant/Research Support James Chappell, MD, PhD, Merck: Grant support for etiologic studies of acute respiratory illness in hospitalized children, Amman, Jordan Natasha B. Halasa, MD, CSL-Seqirus: Advisor/Consultant|Merck: Grant/Research Support

